# Effects of Somatic Mutations in the C-Terminus of Insulin-Like Growth Factor 1 Receptor on Activity and Signaling

**DOI:** 10.1155/2012/804801

**Published:** 2012-06-14

**Authors:** Barbara P. Craddock, W. Todd Miller

**Affiliations:** Department of Physiology and Biophysics, School of Medicine, Stony Brook University, Stony Brook, NY 11794, USA

## Abstract

The insulin-like growth factor I receptor (IGF1R) is overexpressed in several forms of human cancer, and it has emerged as an important target for anticancer drug design. Cancer genome sequencing efforts have recently identified three somatic mutations in IGF1R: A1374V, a deletion of S1278 in the C-terminal tail region of the receptor, and M1255I in the C-terminal lobe of the kinase catalytic domain. The possible effects of these mutations on IGF1R activity and biological function have not previously been tested. Here, we tested the effects of the mutations on the *in vitro* biochemical activity of IGF1R and on major IGF1R signaling pathways in mammalian cells. While the mutations do not affect the intrinsic tyrosine kinase activity of the receptor, we demonstrate that the basal (unstimulated) levels of MAP kinase and Akt activation are increased in the mutants (relative to wild-type IGF1R). We hypothesize that the enhanced signaling potential of these mutants is due to changes in protein-protein interactions between the IGF1R C-terminus and cellular substrates or modulators.

## 1. Introduction

The human genome encodes approximately 90 tyrosine protein kinases [1]. A common characteristic of these enzymes is that they are normally tightly regulated in unstimulated cells. Stimulation (e.g., by binding of a growth factor to the extracellular domain of a receptor tyrosine kinase) leads to a rapid, transient increase in tyrosine kinase activity. *Constitutive* activation of tyrosine kinases, however, is often observed in cancer cells. Genes that are causally implicated in human cancer frequently encode protein kinase catalytic domains [2]. Most oncogenic tyrosine kinases contain activating mutations and are dominant at the cellular level [2, 3].

The human insulin-like growth factor 1 receptor (IGF1R) is a heterotetramer containing two extracellular alpha subunits and two transmembrane beta subunits [4]. Binding of the ligand (IGF1) to the alpha subunits triggers a conformational change that leads to autophosphorylation of the intracellular kinase domains in the beta subunits [5]. Autophosphorylation greatly enhances the activity of the IGF1R catalytic domain [6]. The signal is propagated through the PI 3′-kinase and MAP kinase pathways to promote proliferation and cell survival. In the unstimulated state, the basal activity of the IGF1R receptor is suppressed by autoinhibitory interactions between the activation loop and other residues in the kinase domain [6–8] and between the kinase domain and the juxtamembrane region [9].

Deregulated IGF1R kinase activity has been linked to human cancer [10–12]. Studies in cell culture systems have shown that overexpression of IGF1R can lead to morphological transformation, while interference with IGF1R reverses the transformed phenotype [13]. IGF1R is overexpressed in numerous solid tumors as well as in multiple myeloma [11, 12]. The cell survival function of IGF1R appears to be critical in these tumors, as inhibition of IGF1R can induce apoptosis. A number of therapeutic approaches are currently being explored to interfere with IGF1R signaling in cancer cells, including RNA interference, receptor antibodies, and small molecule kinase inhibitors [11, 12]. Several mechanisms have been reported to lead to IGF1R activation in cancer cells. Increased transcription of the IGF1R gene has been shown to result from loss of tumor suppressor genes, such as, p53 or from the action of other oncogenes [14]. Loss of imprinting (LOI) of IGF2 is an epigenetic alteration found in many colorectal and other tumors [15]. To date, no activating IGF1R mutations have been identified in cancers.

Recent gene sequencing efforts have catalogued hundreds of somatic mutations in the coding regions of potential cancer genes. These mutations comprise both “driver” mutations (which confer a growth advantage and are causally connected to the development of cancer [16]) and “passenger” mutations, which do not contribute to the development of cancer. Screening for somatic mutations in kinase genes identified two mutations in the gene encoding IGF1R that led to aminoacid changes: A1347V (from lung squamous cell carcinoma) and an in-frame deletion of S1278 (from renal clear cell carcinoma) [16]. In a separate study, a M1255I mutation was identified in lung adenocarcinoma [17]. The effect of these mutations, if any, on the biological function of IGF1R has not been tested. M1255 falls in the C-terminal lobe of the tyrosine kinase catalytic domain, while S1278 and A1347 lie in the C-terminal portion of the receptor. We report the effects of the mutations on the* in vitro* biochemical activity of IGF1R, as well as on the major IGF1R signaling pathways in mammalian cells.

## 2. Materials and Methods

### 2.1. Western Blotting Analysis of IGF1R Activity and Signaling

Mutant forms of IGF1R were generated by site-directed mutagenesis (QuikChange Kit, Stratagene) on the expression vector pBPV-IGF1R [5]. R-cells are a murine fibroblast cell line deficient in IGF1R [18]. One million R-cells were plated onto 10 cm tissue culture dishes and grown to 50% confluency in DMEM plus 4500 mg/L glucose (Fisher/Cellgro), 10% heat inactivated fetal bovine serum (VWR), 1X antibiotic/antimycotic (Fisher/Cellgro) and 50 ug/mL G418 (Sigma). R-cells were transfected with the pBPV-IGF1R constructs using TransIT polyamine transfection reagent (Mirus) according to the manufacturer's instructions. After 24 hours, the transfection mixture was replaced with starvation media containing DMEM, 1000 mg/L glucose (Invitrogen), 1X antibiotic/antimycotic (Fisher/Cellgro), 0.5% BSA, 50 ug/mL G418, and 50 ug/mL holotransferrin (Sigma). After 16 hours of starvation at 37°C and 5% CO_2_, the cells were stimulated for 10 minutes at 37°C with 40 ng/mL IGF1 (Calbiochem). Unstimulated controls were included in each set of experiments. The cells were washed with 4°C PBS (Sigma) and lysed in 1 mL of lysis buffer containing 25 mM Tris-HCl (pH 8), 2 mM EDTA, 140 mM NaCl, 1% NP40, 0.5 ug/mL leupeptin, 0.5 ug/mL aprotinin, and 2 mM activated sodium orthovanadate. After clearing the lysates by centrifugation, *2 *μ*g* of anti-IGF1R antibody (clone JBW902, Millipore) was added to the lysates for 1 hour at 4°C, followed by the addition of *60 *μ*L* protein-A-conjugated agarose beads (Sigma). The tubes were rocked gently overnight at 4°C. The following day the beads were washed 5 times with cold lysis buffer. The precipitated proteins were analyzed on 10% SDS polyacrylamide gels and transferred to PVDF membranes (Millipore). The blots were probed with anti IGF1R antibody (Millipore), then stripped and reprobed with anti-IGF1R (pYpY1135/1136) (BioSource). The blots were visualized using the SuperSignal West Femto Maximum Sensitivity substrate system (Pierce). Densitometry and analysis were performed using ImageJ version 1.42 and Prism version 4.

In a separate set of experiments, the IRS-1 receptor was immunoprecipitated in a similar manner using anti IRS-1 (Millipore). Cells were stimulated with 10 ng/mL IGF1 (the lower concentration was used to eliminate any contribution from insulin receptor). The blots were probed with antiphosphotyrosine (4G10) antibody. To analyze Akt and MAP kinase activation, 50 ug of lysates was analyzed on 10% SDS polyacrylamide gels, transferred to PVDF membranes and probed with antiphospho-AKT and antiphospho-MAP kinase antibodies. Membranes were stripped and reprobed with Akt and MAP kinase antibodies for normalization (Cell Signaling). To analyze the association of IGF1R and RACK1, lysates (2 mg) were immunoprecipitated with *4 *μ*g* of anti IGF1R C20 antibody (Santa Cruz) and *60 *μ*L* protein-A-conjugated agarose beads. After analysis on 10% SDS polyacrylamide gels and transfer to PVDF membranes, the blots were probed with anti RACK1 (BD Transduction) and anti IGF1R (clone JBW902) antibodies.

### 2.2. Expression and Purification of Proteins

DNA sequences encoding IGF1R residues 930–1337 (the complete cytoplasmic domain of IGF1R) had previously been subcloned into pFastBac Htb (Life Technologies, Inc.) [9]. Site directed mutagenesis was performed to generate the M1255I, *Δ*S1278, and A1347V mutants. After confirming the mutations by DNA sequencing, recombinant virus was generated using the Bac-to-Bac system (Life Technologies, Inc.). The virus was then used to produce protein by infection of *Spodoptera frugiperda* (Sf9) cells. One billion Sf9 cells were infected with high-titer virus and harvested after 72 hours. The cells were lysed in a French pressure cell in lysis buffer containing 50 mM Tris-HCl (pH 8.5), 5 mM EDTA, 1% Triton-X 100, 5 mM 2-mercaptoethanol, 0.5 ug/mL aprotinin, 0.5 ug/mL leupeptin, 2 mM PMSF, and 2 mM activated sodium orthovanadate. The clarified lysate was loaded onto a 5 mL column of Ni-NTA resin (Qiagen) which had been preequilibrated with buffer A (20 mM Tris-HCl pH 8.5, 500 mM KCl, 10% glycerol). Imidazole (20 mM) and 2-mercaptoethanol (5 mM) were added to buffer A after the column had been equilibrated. The column was washed with 10 column volumes of buffer A containing imidazole (20 mM) and 2-mercaptoethanol (5 mM), followed by 2 column volumes of buffer B (20 mM Tris-HCl pH 8.5, 1 M KCl, 5 mM 2-mercaptoethanol, 10% glycerol), then 10 column volumes of buffer A. The IGF1R cytoplasmic domain was eluted with buffer C (20 mM Tris-HCl pH 8.5, 100 mM KCl, 100 mM imidazole, 5 mM 2-mercaptoethanol, 10% glycerol). The fractions were analyzed on 10% SDS-polyacrylamide gels, and the peak fractions were pooled and stored in aliquots at −80°C in 40% glycerol.

### 2.3. Protein Kinase Assays

A continuous spectrophotometric assay [19] was used to analyze autophosphorylation of the purified proteins. The experiments were performed at 30°C in buffer containing 100 mM Tris-HCl (pH 7.5), 10 mM MgCl_2_, 1.5 mM phosphoenolpyruvate, 201 U/mL pyruvate kinase, 230 U/mL lactate dehydrogenase, and 0.5 mM NADH. The concentration of the enzyme was *2 *μ*M* and the concentration of ATP was 1 mM. For peptide phosphorylation experiments, the enzymes were prephosphorylated by incubation with 5 mM ATP for 15 minutes at room temperature prior to the assays. Phosphorylation of the peptide KKEEEEYMMMMG was measured using the phosphocellulose paper binding assay [6]. The final concentration of the enzymes in these assays was 800 nM, the concentration of the ATP was 1 mM, and the concentration of the peptide was 1 mM.

## 3. Results

**Figure 1:**
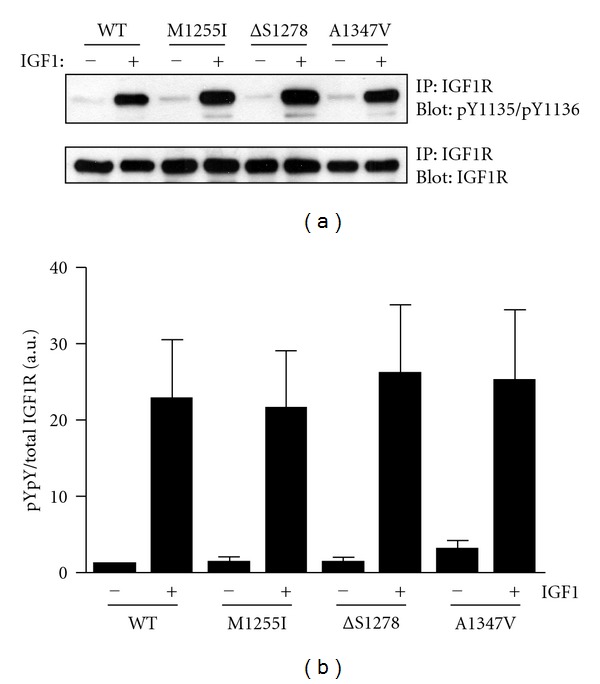
Cellular autophosphorylation activity of wild-type and mutant receptors. R-cells were transfected with wild-type or mutant forms of IGF1R. After overnight starvation, cells were treated with or without IGF1 for 10 minutes. (a) Top panel: IGF1R was immunoprecipitated and analyzed by anti-pY1135/pY1136 Western blotting. Bottom panel: the membrane was stripped and reprobed with anti-IGF1R antibody. (b) Densitometric analysis was carried out, and the pY1135/pY1136 signal was normalized to the total IGF1R signal. The bar graph shows results from three experiments (mean ± standard deviation). The results were analyzed by Student's *t*-test, and no statistically significant differences were observed between wild type and mutants (*P* > 0.1).

We expressed full-length IGF1R harboring the M1255I, *Δ*S1278, or A1347V mutations in fibroblasts derived from IGF1R-deficient mice (R-cells). For comparison, we expressed wild-type IGF1R in the same cell background. As an initial check of the activity of the receptors, we measured autophosphorylation at Y1135/Y1136. Tris-phosphorylation of IGF1R at Y1131/Y1135/Y1136 in the activation loop produces a large increase in kinase activity [6]. We immunoprecipitated IGF1R from the R-cells, separated proteins by SDS-PAGE and analyzed them by Western blotting with a phospho-specific antibody to pY1135/pY1136 (Figure 1). Membranes were stripped and reprobed with an anti-IGF1R antibody; normalization of the pY1135/pY1136 signal with respect to total IGF1R revealed no significant change in levels of autophosphorylation. Thus, the mutations did not drastically affect the intrinsic tyrosine kinase activity of IGF1R.

**Figure 2:**
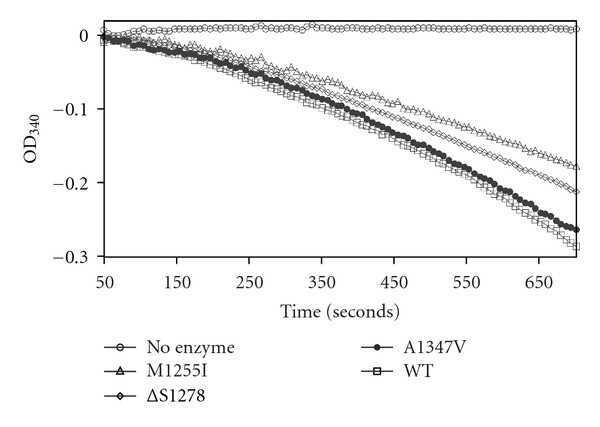
*In vitro* autophosphorylation activity of purified wild-type and mutant cytoplasmic domains. Enzymes (*4 *μ*M*) were incubated with 1 mM ATP, and autophosphorylation was measured using the coupled spectrophotometric assay. Data were recorded every 6 seconds.

We previously reported the expression and purification of the entire cytoplasmic portion of IGF1R (i.e., juxtamembrane, kinase, and C-terminal domains) from insect cells using a baculovirus expression vector [9]. We generated recombinant baculoviruses encoding the M1255I, *Δ*S1278, and A1347V mutant forms of IGF1R. We infected *Spodoptera frugiperda* cells with mutant or wild-type IGF1R baculoviruses, harvested cells after three days of infection, and purified the proteins (Supplemental Figure 1) Supplementary materials are available online at doi:10.1155/2012/804801. We examined autophosphorylation activity of the purified receptors using a coupled spectrophotometric assay [19]. The enzymes were incubated with ATP in the absence of an exogenous substrate. All forms of IGF1R tested were active in the *in vitro* autophosphorylation assay (Figure 2). As observed previously for wild-type IGF1R [6], the enzyme progress curves were biphasic, due to the fact that autophosphorylation increases the enzymatic rate. The rates of wild-type and A1347V IGF1R were comparable, while the rates of the *Δ*S1278 and M1255I mutant forms were somewhat slower.

**Figure 3:**
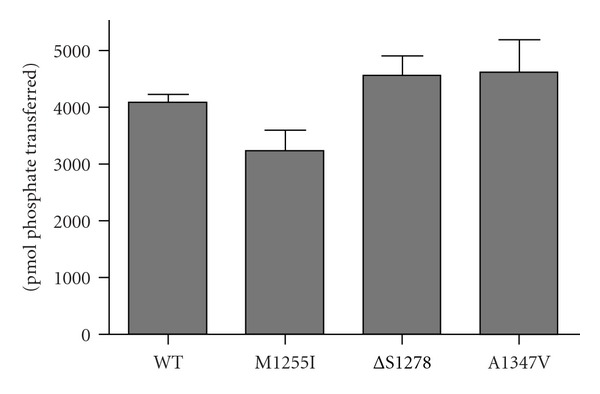
Peptide phosphorylation by purified wild-type and mutant cytoplasmic domains. The preautophosphorylated proteins (800 nM) were incubated with 1 mM peptide (KKEEEEYMMMMG) and 1 mM ATP, and activity was measured using the phosphocellulose paper binding assay. Reactions proceeded for 20 minutes at 30°C. Assays were conducted in triplicate, and the results are presented as mean ± standard deviation

Next, we examined the ability of the purified receptors to phosphorylate an exogenous substrate. In these experiments, we used a synthetic peptide substrate previously shown to be phosphorylated by IGF1R with good kinetic parameters [6, 20]. To eliminate the effect of autophosphorylation, we first prephosphorylated the proteins by treatment with ATP [9, 21]. The wild-type and mutant forms of IGF1R were all active in this assay (Figure 3). The M1255I mutation, which occurs toward the C-terminal end of the kinase catalytic domain, showed a small reduction in peptide phosphorylation. Taken together, the receptor phosphorylation assays (Figure 1) and the autophosphorylation and peptide phosphorylation assays (Figures 2 and 3) showed that the mutant kinase domains were active but were not hyperactivated relative to wild type.

**Figure 4:**
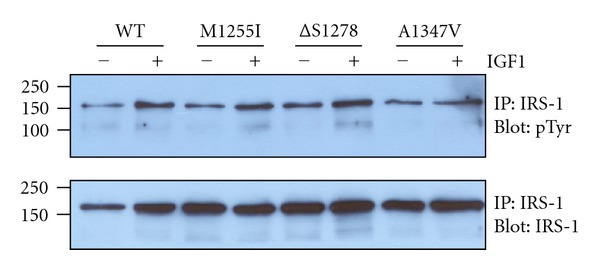
IRS-1 phosphorylation. R-cells were transfected with wild-type or mutant forms of IGF1R. After overnight starvation, cells were treated with or without IGF1 for 10 minutes. Endogenous IRS-1 was immunoprecipitated and analyzed by antiphosphotyrosine Western blotting. The membrane was stripped and reprobed with anti-IRS-1 antibody.

The insulin receptor substrate (IRS) family adaptor proteins are important proximal substrates for the activated insulin and IGF1 receptors [22, 23]. IRS-1 contains N-terminal PH and PTB domains which play important roles in directing the adaptor protein to IR/IGF1R. The PTB domain binds specifically to phosphorylated Y960 in the juxtamembrane region. IRS-1 also contains multiple YMXM motifs which are phosphorylated by the receptor catalytic domains [24]. These phosphorylated sequences provide binding sites for Grb2, phosphoinositide 3′-kinase, Nck, SHP2, and other cellular signaling proteins containing SH2 domains. We expressed wild-type and mutant forms of IGF1R in R-cells and analyzed the overall tyrosine phosphorylation of endogenous IRS-1 by immunoprecipitation and Western blotting with anti-pTyr antibodies. These results showed that the mutant forms of IGF1R were capable of phosphorylating IRS-1 (Figure 4). The levels of phosphorylation were not significantly different for the mutants versus wild type.

**Figure 5:**
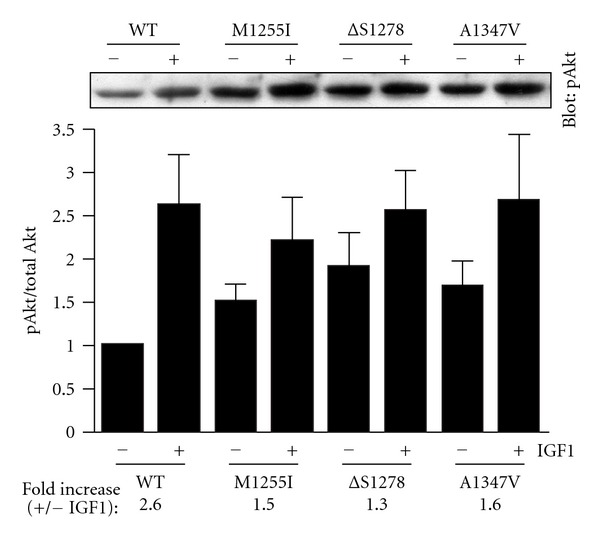
Akt phosphorylation. R-cells were transfected with the indicated forms of IGF1R. After starvation and treatment with IGF1, Akt activity was measured in cell lysates using a phosphospecific antibody. Membranes were stripped and reprobed with anti-Akt antibody. The bar graph shows the ratio of phospho-Akt to total Akt (mean ± standard deviation). The fold increases in the presence of IGF1 are given below the bar graph. A representative phospho-Akt blot is shown above the graph. The results were analyzed by Student's *t*-test, and no statistically significant differences were observed between wild type and mutants (*P* > 0.1).

We examined downstream signaling for wild-type and mutant forms of IGF1R. After transfection with IGF1R expression plasmids, R-cells were serum starved, then stimulated with IGF1 or left unstimulated. We measured Akt activation in cell lysates using an activation state-specific antibody (Figure 5). Western blots were reprobed with total Akt antibody for normalization. Figure 5 shows a representative Western blot, along with a bar graph depicting a summary of all the experiments. Akt activation in the presence of IGF1 was similar for the various forms of IGF1R tested (Figure 5). The basal, unstimulated levels of Akt activity were 1.5–2-fold higher for the *Δ*S1278, A1347V, and M1255I forms of IGF1R, and the fold increases (stimulated/unstimulated) were consequently lower. 

**Figure 6:**
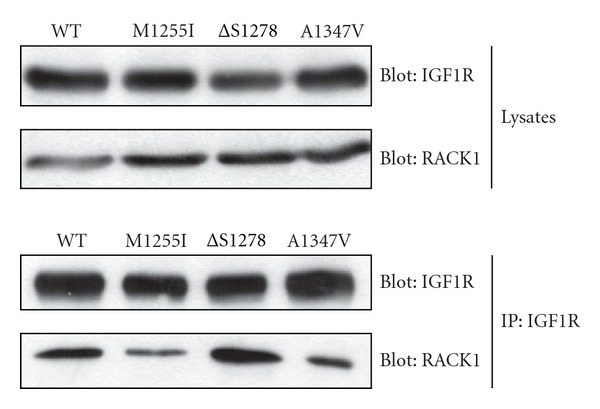
RACK1 association. Wild-type and mutant forms of IGF1R were transfected into R-cells. IGF1R was immunoprecipitated from the cells and analyzed by anti-RACK1 and anti-IGF1R Western blotting.

As an attempt to gain insight into the basis for increased levels of phospho-Akt in the absence of IGF1, we analyzed the association between IGF1R and the RACK1 adaptor protein. RACK1 interacts with IGF1R to negatively regulate the PI 3′-kinase pathway [25]. The association between IGF1R and RACK1 is independent of receptor activation, but a Ser-to-Ala mutation at S1278 of IGF1R was reported to block RACK1 binding [25]. Because one of the cancer-associated mutations involved the same residue (*Δ*S1278), we measured the coimmunoprecipitation of endogenous RACK1 with wild-type and mutant forms of IGF1R in R-minus cells. Association with RACK1 was unaffected by the *Δ*S1278 and other mutations (Figure 6).

**Figure 7:**
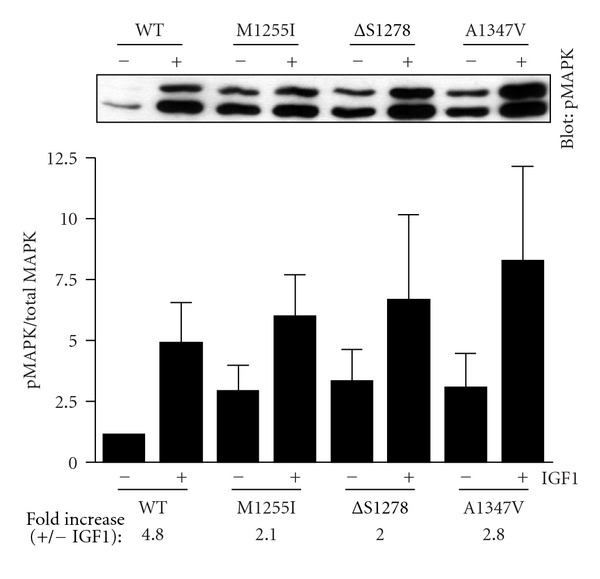
MAP kinase activation. R-cells were transfected with the indicated forms of IGF1R. After starvation and treatment with IGF1, MAP kinase activity was measured in cell lysates using a phosphospecific antibody. Membranes were stripped and reprobed with anti-MAPK antibody. The bar graph shows the ratio of phospho-MAPK to total MAPK (mean ± standard deviation). The fold increases in the presence of IGF1 are given below the bar graph. A representative phospho-MAPK blot is shown above the graph. The results were analyzed by Student's *t*-test, and no statistically significant differences were observed between wild type and mutants (*P* > 0.1).

Next, we carried out similar experiments to measure activation of MAP kinase in R-cells expressing wild-type or mutant forms of IGF1R. MAPK activity was measured by Western blotting with a phosphospecific antibody, and total MAPK levels were assessed by reprobing with a MAPK antibody. As expected, IGF1 stimulation led to an increase in MAP kinase activation in R-cells expressing wild-type IGF1R. Hormone stimulation of R-cells expressing mutant forms of IGF1R led to levels of MAP kinase activation that were not significantly different from wild type. On the other hand, the basal (unstimulated) levels of MAP kinase activation were 2.5-3-fold higher for the mutants that for wild type (Figure 7). Thus, the fold increases (stimulated/unstimulated) for the M1255I, *Δ*S1278, and A1347V mutants were 2.1, 2.0, and 2.8, respectively, compared to 4.8 for wild type. These results, and the similar findings for Akt signaling (Figure 5), suggest that the regulatory interactions that normally keep IGF1R activity in check were partially disrupted in the case of the cancer-associated mutants.

## 4. Discussion

For several receptor tyrosine kinases, biochemical and structural studies have implicated the C-terminal portion of the cytoplasmic domain of several RTKs in kinase signaling. The deletion of the C-terminal residues of ErbB2 increased receptor kinase activity as well as transforming ability [26]. A peptide containing the C-terminus of PDGFR *β* receptor inhibited its kinase activity [27]. The crystal structure of the Tie2 RTK has been solved with an intact C-terminal tail [28]. The Tie2 C-terminal tail interacts with the C-lobe of the kinase catalytic domain and ends near the substrate binding site. Deletion of the Tie2 C-terminal tail increased receptor autophosphorylation and kinase activity, lending support to a model in which the tail is involved in kinase regulation [29]. 

Current evidence suggests that the C-termini of IR and IGF1R do not play a direct role in controlling the activation kinetics of the catalytic domains [30, 31]. On the other hand, the C-terminal domain is involved in downstream cellular signaling. A chimeric IGF1R containing the C-terminal domain of IR was more potent in stimulating glycogen synthesis than wild-type IGF1R [32], suggesting that biological specificity resides (at least in part) in the C-terminus. This idea has been reinforced by studies demonstrating differential binding and phosphorylation of cellular proteins mediated by the C-termini of IGF1R and IR. CEACAM1 is an example of a protein that binds specifically to the C-terminus of IR [33], 14-3-3 proteins and IIP-1 bind selectively to the IGF1R C-terminus [34, 35], and RACK1 interacts with both C-termini [25]. Protein-protein interactions mediated by the C-terminus play a role in downstream IGF1R signaling. In particular, deletion of the IGF1R C-terminus increased the ability of the receptor to promote cell survival [36], suggesting that the C-terminus has a negative regulatory role. Consistent with this idea, tumor cell apoptosis was increased by stable expression of a myristoylated IGF1R C-terminus [37] or by introduction of a synthetic 17-aminoacid peptide from the C-terminus [38]. Point mutations in the IGF1R C-terminus inhibit cell survival [39] and anchorage-independent growth [40], while deletion of the C-terminus blocks receptor ubiquitination [31]. 

Two of the cancer-associated IGF1R mutations studied in this paper (*Δ*S1278 and A1347V) fall in the C-terminus and one (M1255I) is in the C-terminal lobe of the catalytic domain. Consistent with prior mutational analyses of the IGF1R C-terminus, the cancer-associated mutations did not significantly affect full-length receptor autophosphorylation in cells (Figure 1). Similarly, the purified cytoplasmic domains retained their autophosphorylation activity *in vitro* and their ability to phosphorylate exogenous peptide substrates (Figures 2 and 3). Phosphorylation of the receptor-proximal substrate IRS-1 also appeared to be unaffected by the mutations (Figure 4). For comparison, a mutagenic analysis of the IGF1R/IR juxtamembrane regions showed that these residues exert direct negative control over receptor phosphorylation and activity [9].

Because previous studies suggested both positive and negative roles of the IGF1R C-terminus in cell signaling, it was not clear what the predicted effects of the cancer-associated mutations would be. While the mutant forms of IGF1R showed no difference from wild-type with respect to the maximal levels of MAP kinase and Akt activation, basal levels of activity were higher (Figures 5 and 7). These results are consistent with a model in which the IGF1R C-terminus plays an inhibitory role in signal transduction, and the mutations partially destabilize the inhibited conformation, leading to enhanced activity. Although the effects observed were modest, these mutations represent the first activating mutations of IGF1R identified in human cancers.

The mechanism for increased IGF1R activity is not clear at present. The mutations studied here do not fall within the sequence (1312–1328) included in the inhibitory synthetic peptide which was previously shown to promote tumor cell apoptosis [38]. The alterations in MAP kinase and Akt signaling are likely due to changes in protein-protein interactions with the IGF1R C-terminus. In principle, the mutations might increase binding of a positive mediator of IGF1R signaling or block binding of an inhibitory protein factor. One candidate of the latter type is the RACK1 adaptor protein. Overexpression of RACK1 inhibits IGF1R-mediated Akt phosphorylation [25]. RACK1 binding requires S1278 in the IGF1R C-terminus, since a Ser-to-Ala mutation eliminates binding. Although one of the cancer-associated mutations is a deletion of this same residue, RACK1 binding was unaffected in our experiments. Future experiments will be aimed at identifying IGF1R-interacting partners that are altered in the cancer-associated variants.

## Supplementary Material

Supplemental Figure 1: Purification of the cytoplasmic domains of wild-type IGF1R and the cancer-associated mutants. The proteins were analyzed by SDS-PAGE with Coomassie staining. The arrowhead indicates the position of IGF1R. The lower-molecular weight contaminant is a degradation product [9].
