# Supplementary material for: Effects of Somatic Mutations in the C-Terminus of Insulin-Like Growth Factor 1 Receptor on Activity and Signaling
Source: J Signal Transduct. 2012 Jun 14;2012:804801. doi: 10.1155/2012/804801 (PMC3384887; doi:10.1155/2012/804801)
Supplement: Supplementary file 1 — Supplemental Figure 1: Purification of the cytoplasmic domains of wild-type IGF1R and the cancer-associated mutants. The proteins were analyzed by SDS-PAGE with Coomassie staining. The arrowhead indicates the position of IGF1R. The lower-molecular weight contaminant is a degradation product [9]. [file 804801.f1.pdf]

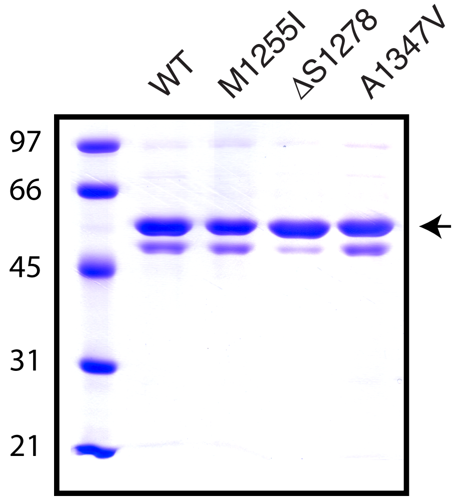

Supplemental Figure 1

Purification of the cytoplasmic domains of wild-type IGF1R and the cancer-associated mutants. The proteins were analyzed by SDS-PAGE with Coomassie staining. The arrowhead indicates the position of IGF1R. The lower-molecular weight contaminant is a degradation product [9].
